# Pregnant Women’s Knowledge of Pelvic Floor and Related Dysfunctions: A Scoping Review

**DOI:** 10.3390/healthcare13080847

**Published:** 2025-04-08

**Authors:** Konstanze Weinert, Claudia F. Plappert

**Affiliations:** Section of Midwifery Science, Institute of Health Sciences, University of Tuebingen, 72076 Tuebingen, Germany; claudia.plappert@med.uni-tuebingen.de

**Keywords:** pregnancy, pregnant women, pelvic floor, pelvic floor disorders, health knowledge, awareness, attitudes

## Abstract

Pregnancy and childbirth can have far-reaching effects on women’s pelvic floor health. It is important to educate pregnant women about pelvic floor health and potential birth-related pelvic floor (PF) changes as part of continuous midwifery care. This scoping review aims to identify the current state of research on knowledge and knowledge gaps in pregnant women regarding the PF and PF dysfunction (PFD) in order to derive conclusions and recommendations for midwifery care and midwifery science. This review follows the Arksey and O’Malley framework and the PRISMA Statement. The literature search was conducted on databases PubMed, CINAHL, and Web of Science using various search terms and defined inclusion criteria. Eleven articles were included. The results show a considerable context-related knowledge deficit among pregnant women, with a high prevalence of PFDs such as urinary incontinence (UI), fecal incontinence (FI), or prolapse symptoms (POP). All contributions emphasize the importance of improved specific education and health advice regarding the PF and PFD for pregnant women to close knowledge gaps and promote sustainable PF health. Demands are made on the professional group ‘midwife’, which emphasize the importance of specific and target group-appropriate educational concepts on the subject of the PF and PFD.

## 1. Introduction

Pregnancy, childbirth, and the postpartum period are significant events in a woman’s life that are accompanied by profound physiological, psychological, and emotional changes. In addition to these changes, women are exposed to increased health risks, including a higher likelihood of developing pelvic floor dysfunction (PFD) such as urinary incontinence (UI), fecal incontinence (FI), or pelvic organ prolapse (POP) [1,2]. The prevalence of urinary incontinence during pregnancy is considerable; in the first trimester, 8–10% of women are affected, rising to 23–32% by the time of delivery. After birth, the prevalence remains high; up to 36% report UI after 6–13 weeks, 31% after 6 months, and even 51% after one year postpartum [3,4]. Even long-term data indicate a persistent burden; around 38% of women report UI 20 years after giving birth [5]. Existing or persistent urinary incontinence during pregnancy and in the early postpartum period has been identified as a significant risk factor for long-term incontinence up to 12 years later [6]. PFDs can have a significant impact on women’s quality of life. The associated consequences include mental illnesses such as depression and anxiety, sexual dysfunction, limited social participation, and restrictions on physical activity [7]. Due to the intimate and often shameful nature of the subject, many affected women are reluctant to ask questions or seek support. Insufficient information from healthcare professionals or midwives contributes to postpartum PFD being seen as a normal and expected outcome of labour, leading to underdiagnosis and inadequate treatment [8,9]. Particularly concerning is that pregnant women know little about the proven preventative effects of pelvic floor muscle training (PFMT). Structured and supervised PFMT, which includes alternating contraction and relaxation of the pelvic floor muscles and pelvic floor awareness exercises, has been shown to be effective in reducing the risk of postpartum UI and FI, particularly in continent pregnant women [10,11]. Nonetheless, adherence to regular PFMT during pregnancy remains low [10,12,13]. This underlines the importance of sensitising pregnant women to the health of the pelvic floor and possible changes in pelvic floor function (PFF) caused by pregnancy or childbirth. It also emphasises the need to provide them with comprehensive information about prevention strategies and how to implement them in everyday life. In Germany, midwives play a central role in promoting pelvic floor health during pregnancy and in the postnatal period. Counselling and guidance on protecting and strengthening the pelvic floor are among the core tasks of midwives, who therefore bear a great responsibility for maintaining perineal health [14,15]. Effective and targeted counselling requires knowing what pregnant women know—or do not know—about the anatomy and function of the pelvic floor and about possible pregnancy-related PFDs.

The aim of this review is therefore to examine the current state of research on the knowledge and health literacy of pregnant women in relation to pelvic floor anatomy, function, and dysfunction. By identifying existing knowledge and gaps, this review provides recommendations for midwifery science and the development of appropriate counselling and education strategies.

## 2. Methods

This form of scoping review is suitable for the existing research question, as this is a relatively new field of research within midwifery science, and existing evidence is to be presented and structured [16]. This paper followed the basic work by Arksey and O’Malley [17], the explanations by Levac et al. [18], the PRISMA 2020 Statement [19,20,21,22], and further recommendations by von Elm et al. [16]. The priori protocol was not registered. Two researchers were involved in the following steps and phases. The following steps were derived and completed: phase 1, identifying the research question; phase 2, identifying relevant studies; phase 3, selecting the studies; phase 4, charting the data; and finally, phase 5, collecting, summarizing, and reporting the results [16,17,18].

Phase 1: Identification of the research question

The research question was formulated according to the PCC framework, which stands for population, concept, and context [16]. The aspects included are listed in Table 1. The resulting research question is: ‘What do pregnant women know about their pelvic floor and the impact of pregnancy on potential pelvic floor dysfunction?’ The aim of the review is to map the available evidence to gain an overview of the following questions:

- What ideas do pregnant women have about PF anatomy and PFF?
- What basic knowledge is available on PFD in the context of pregnancy and birth?
- What is the level of knowledge on the prevention of PFD or on measures to restore PF health in the case of pregnancy-associated PFD?
- What factors promote an improvement in the level of knowledge of pregnant women regarding the PF and possible pregnancy-associated PFDs?
- Which medical services are sought by pregnant women with PFDs?

Phase 2: Identifying relevant studies

A comprehensive search strategy was used to identify the relevant literature. Using specific keywords and search terms, placeholders, and Boolean operators ‘OR/AND’ as well as truncation (*) in the word stem “pregnancy”, various databases were searched for topic-specific articles. The literature search was carried out in consultation and in cooperation with the Medical Library of the University Hospital Tuebingen. The three large and medically relevant databases PubMed (Medline), CINAHL, and Web of Science were selected. Table 2 shows the search strategies described with the corresponding search terms:

Phase 3: Study selection

The first step in selecting relevant studies was to remove duplicates and short meeting abstracts after importing all the studies searched for into a Microsoft Excel file. The remaining studies were imported into the EndNote 21 software. The articles were then checked for suitability of the title and abstract. After excluding articles that did not fit the research question, the remaining articles were checked for suitability by means of full-text screening and examination of inclusion criteria for the final inclusion of the relevant study articles in this scoping review. The literature search and selection took place between July and August 2024 and was carried out by two independent researchers. The inclusion or exclusion of various articles was discussed in personal conversations and discursive consensus. In order to obtain a comprehensive overview of the topic of this literature review, literature from January 2004 to 31. July 2024 was included. The type of study was not restricted to implementing a search strategy that was as sensitive as possible. Publications in English and German were considered. Associated inclusion criteria are shown in Table 3.

Type of Sources

A critical appraisal of studies is not intended in a scoping review [16,18]. The scoping review approach chosen here allows for the exploration of research questions even in areas where only limited literature is available. A formal critical appraisal might otherwise exclude studies that offer valuable insights relevant to the research question. Thus, all types of studies focusing on the knowledge of pregnant women on the PF and PFD were included. A national and international overview is provided by German and English language publications from the last 20 years that were included in the screening process. Data were only extracted if a German or English translation of the full text was available. Short articles, such as published poster presentations, were not included. Furthermore, studies that investigated the knowledge of the PF or PFD in non-pregnant women were not included. Further exclusion criteria were publications before January 2004, exclusive research subject pelvic floor muscle training during pregnancy or postpartum with or without forms of incontinence, and sexual behaviour during pregnancy. Although a sensitive search was conducted from 2004 onwards, relevant publications were available between 2016 and 2024.

Phase 4: Charting the data

The data extraction was based on the objective and research question of this scoping review, which led to a descriptive summary of the results. The following characteristics were extracted from the literature and summarized in a Table: author, year, title, country, objective, population, methods (Table 4), and relevant outcomes and recommendations (Table 5).

Phase 5: Collecting, summarizing, and reporting the results

At this point, information was extracted from eleven studies conducted in nine different countries. The research question specified in Step 1 could be answered on the basis of the study characteristics defined in Step 4. In this way, important findings can be presented with regard to the research question of this literature review.

## 3. Results

**Figure 1:**
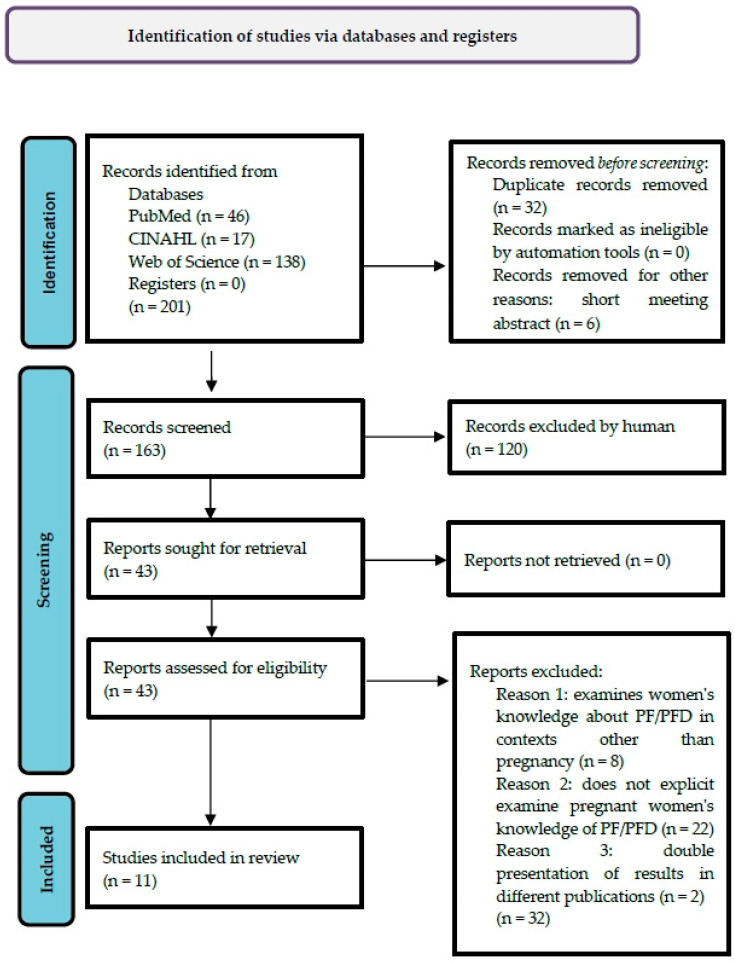
PRISMA Flow Diagram.

The literature search identified 201 relevant records in three databases, of which 32 duplicates and 6 short meeting abstracts were removed. After reviewing n = 163 records for title and abstract, n = 43 remaining articles were identified for subsequent full-text screening for the inclusion criteria presented. This was followed by the exclusion of n = 32 articles that did not meet the inclusion criteria. Thus, n = 11 articles were included in this literature review. The search process is illustrated in the PRISMA flow diagram [22] (Figure 1):

### 3.1. Characteristics of Included Sources of Evidence

All sources were scientific publications in scientific journals. The included articles were published between 2016 and 2024 and were from nine different countries [23,24,25,26,27,28,29,30,31,32,33]. Two each of the eleven integrated studies originated from the USA and Turkey. In addition, one study each from the UK, Australia, Malaysia, Singapore, Israel, Brazil, and Ethiopia was integrated. What all the studies had in common was that they investigated pregnant women’s knowledge of PF, PFF, and PFD. Ten of the eleven studies were conducted in a quantitative design, of which two were multicentre-cross-sectional surveys [23,24] and eight were cross-sectional surveys [25,26,27,28,29,30,31,32]. Seven out of ten studies [23,25,26,29,30,31,32] used the validated 24-item Prolapse and Incontinence Knowledge Questionnaire (PIKQ) with outcome measures of knowledge in UI and POP as the baseline instrument. This provides three response options for items in each assigned subscale. The correct answer ‘Agree’ is scored as 1, the answers ‘Disagree’ and ‘Not sure’ are scored as 0. A score of at least 10 out of 12 on the UI subscale and 6 out of 12 on the POP subscale is considered good knowledge competence [34]. In two studies, the additional instrument of the validated Pelvic Floor Distress Inventory-20-Questionnaire (PFDI-20) was used with outcome measures for the diagnosis and severity of PFD, such as UI, POP, and FI [23,25]. Here too, a total score (0 to 300 points) from 20 questions in 3 dimensions (UI/POP/FI) reflects the individual burden of PFD. The higher the score, the higher the individual symptomatic burden [35]. One study used a self-designed validated questionnaire based on the PIKQ [24]. Two further studies supplemented the PIKQ with additional questions [30,31]. One study [29] used the validated Urinary Incontinence Attitude Scale (UIAS) [36] as a second instrument, which measures attitudes towards UI. Another study utilized a specially designed, validated, and piloted questionnaire with items that measured awareness of PFMs and knowledge of PFMs and PFMTs, and explored the role of PFMs as a key function in the prevention of UI [27]. Another study utilized a specially designed, validated, and piloted questionnaire with outcome measures regarding knowledge of PFD (UI/POP/FI) and how they relate to pregnancy and childbirth [28]. Only one study was conducted using a qualitative design. The focus here was on conducting a semi-structured interview on knowledge about the PF and the reproductive system, as well as a graphic representation of this by the study participants [33].

### 3.2. Literature Synthesis

The detailed study description and its results within the literature synthesis are listed in Table 4 and Table 5. The results (Table 5) represent the key aspects with regard to the knowledge of pregnant women in the context of the PF and PFD, which illustrate the challenges and opportunities for midwifery science.

The following section summarizes the findings of the included studies based on the initially formulated aspects of the research question and addresses both commonalities and discrepancies among the studies.

#### 3.2.1. Knowledge and Ideas About Pelvic Floor Anatomy and Pelvic Floor Functions

Three studies show that pregnant women have little knowledge of PF anatomy and the functions of the various PFMs [27,31,33]. While anatomical structures of the reproductive system, including the uterus, fallopian tubes, and ovaries, are known, the PMF as part of the female body is difficult to visualize and comprehend due to its hidden structure [33]. For example, Hill et al. found that only 5.4% of the study population were able to describe the correct PF anatomy. Only 54% of the study participants knew that the PFMs run around the bladder opening, and a further 20% were unable to categorize the function of the PFMs [27]. Toprak Celenay et al. show similar results with regard to knowledge of PFM functionality; approximately one third of the study population was unable to establish a link between intact PFM function and PF health [31]. Kammers et al. showed that the pregnant women involved had a good visualization of the anatomical structures of the organs of the female reproductive system, but the PFMs were judged to be difficult to comprehend. These appear to be hidden as muscular structures and are associated with the sexual experience of women, which is generally taboo, but especially during pregnancy [33].

#### 3.2.2. Basic Knowledge of Pelvic Floor Dysfunctions (UI, POP, FI) in the Context of Pregnancy and Birth

Overall, pregnant women’s knowledge of PFDs such as UI and POP is low [23,24,25,26,27,28,29,30,31,32]. This also applies to FI [24,28]. UI during pregnancy sometimes appears to be considered ‘normal’ by women [27,29], and UI or POP is also categorized as unproblematic or not necessarily requiring treatment [31]. Although the general level of knowledge with regard to PFD can be categorized as low, it is apparent that there is more knowledge about the pathogenesis and perception of UI compared to POP [24,26,28,31,32] or POP and FI [24,28]. Geynisman-Tan et al. reported a mean knowledge score on UI of 7.9 out of 12 points (66% ± 12) and on POP of 4.9 out of 12 points (41% ± 17) [26]. Liu et al. showed an increased knowledge of UI (46.2 ± 0.3%), followed by FI (39.8% ± 0.3) and POP (35.3% ± 0.3) [28]. O’Neill et al. reported that knowledge of UI (63%) was the highest, followed by POP (36%) and FI (35%) [24]. Toprak Celenay et al. also showed an increased knowledge of UI followed by POP. The mean PIKQ-UI score was 6; the mean PIKQ-POP score was 5 out of 12 points [31]. Similarly, Yohay et al. showed an average higher knowledge of UI (7.65 ± 2.8) compared to POP (5.32 ± 2) [32].

Individual studies investigated participants’ knowledge of whether the occurrence of PFD postpartum was associated with vaginal birth [23,26,29]. This only appears to be the case to a limited extent. Geynisman-Tan et al. showed that only 62.5% of respondents knew that multiple childbearing was a higher risk for UI, compared to only 43% of respondents who knew that the same was true for an increased risk of POP [26]. Similarly, the study by Mckay et al. shows that 49.7% and 29.2% of the pregnant women included in the study knew that multiple births were a risk factor for UI and POP [23]. Parlas and Bilgic also show that in their study population, only 56.1% of participants considered multiple births to be a risk factor for UI [29].

#### 3.2.3. State of Knowledge on the Prevention of Pelvic Floor Dysfunction or on Measures to Restore Pelvic Floor Health in the Presence of Pregnancy-Associated Pelvic Floor Dysfunction

In the context of preventive or therapeutic PFMT during pregnancy, there is also a low level of knowledge among pregnant women [26,27,28,29,30]. Geynisman-Tan et al. showed that 83% of the study participants (n = 402) knew that UI can be counteracted by PFMT, but only 55% of the study population saw a relationship with POP [26]. Hill et al. described that 40% of the study participants (n = 633) considered UI during pregnancy to be ‘normal’ [27]. Many pregnant women do not realize that PFMT can be helpful in preventing or reducing UI during pregnancy. According to Hill et al., this was the case in one-third of all respondents [27] and in 40% of the study participants for Liu et al. [28]. Parlas and Bilgic reported that in their overall study population (n = 255), 44% did not know to what extent they could prevent UI themselves or how they could treat it. Only 53.3% of participants knew that UI can be prevented or reduced by PMFT. Only 6.7% of participants were already aware of PFMT, 37.2% had heard of PMFE, and 16.5% had performed PFMT for the prevention or management of UI [29]. Hill et al. reported that only 11% of the study cohort performed PFMT during pregnancy, and a further 30% were unsure if they had ever contracted their PFM [27]. This finding is also confirmed by Tennfjord et al. Within their study population (n = 502), there was also a low level of knowledge about preventive or therapeutic PFMT and a low level of implementation of preventive or therapeutic PMFT. Only 26.6% of participants had even heard of PMFT. Compared to multiparous women, first-time mothers had slightly better knowledge and attitudes towards PFMT, while multiparous women had slightly better PFMT practice [30].

#### 3.2.4. Factors That Support an Improvement in Pregnant Women’s Knowledge of the Pelvic Floor and Possible Pregnancy-Associated Pelvic Floor Dysfunction

A number of factors have a favourable effect on the context-related knowledge of pregnant women. Increasing maternal age [28,30,32] and higher levels of education and training [23,24,25,28,30,32] are associated with significantly improved knowledge and understanding of the PF and PFD. A linguistic understanding of the local language can be seen as an important prerequisite for acquiring knowledge [27]. High socio-economic status [28,32], employment [28] and nulliparity [28,30] are also discussed as promoting the acquisition of knowledge. Two studies show that pregnant women who work as healthcare professionals have a higher level of knowledge about UI and POP [23], or significantly higher than pregnant women who do not [32]. Similarly, attending antenatal education (ANE) courses improves [31] or significantly improves [27] knowledge of PFF and PFD. However, ANE does not necessarily appear to be a positive resource for improving knowledge of PFD [24]. Specialist care during pregnancy is associated with better knowledge of PFD [25]. Access to the specialities of urology/urogynaecology is associated with higher knowledge of POP [23] and significantly higher knowledge of UI [32].

The main sources used by pregnant women to acquire knowledge about PFD are the internet [24,27,31] and the media as a superordinate, unspecified domain [30]. Books are an important source of information on pregnancy and childbirth [24] and other information on PFM [27] and PFD [24,31]. Family and friends [24,30,31] as well as medical staff [24,30,31] and midwives [24,27,31] are listed as groups of persons who are consulted by pregnant women to acquire knowledge about PFM and PFD.

#### 3.2.5. Seeking Medical Help for Pregnant Women in the Event of Pelvic Floor Dysfunction

A large number of studies report a high prevalence of PFD in the context of pregnancy and childbirth [23,25,28,29,31]. During pregnancy, UI occurs most frequently, followed by POP and FI [25,28,29,31]. Four studies graded the prevalence of UI, POP, and FI [25,28,29,31]. UI is the most common form of PFD during pregnancy. Farihan et al. reported that 46.1% of n = 424 primiparous and multiparous women developed PFD, with 62.7% developing UI, 41.1% developing symptoms of POP, and 37.8% developing symptoms of FI [25]. Liu et al. also recorded a UI-prevalence of 31.7%, a POP-prevalence of 2.9%, and an FI prevalence of 0.96% in a study population of n = 104 primiparous women [28]. Parlas and Bilgic showed a high UI prevalence of 51% in n = 255 primiparous and multiparous women [29]. Toprak Celenay et al. showed a UI-prevalence of 18.9% and symptoms of POP-prevalence of 3.6% within their study population of n = 241 primiparous women [31]. Despite this relatively high prevalence of PFD in pregnant women, three studies reported that affected pregnant women rarely seek medical help. This applies to pregnant women who have newly developed symptoms of UI, POP, and also FI, or who already had them [28,29,31].

## 4. Discussion

### 4.1. Summary of the Results

The literature synthesis of 11 studies [23,24,25,26,27,28,29,30,31,32,33] shows a lack of knowledge among pregnant women about PF anatomy and PFF [27,31,33] and in the area of possible pregnancy-associated PFDs [23,24,25,26,27,28,29,30,31,32]. In addition, there is little knowledge with regard to the prevention of PFD [26,27,28,29,30] and medical assistance is relatively rarely utilized [28,29,31]. The main sources used by pregnant women to acquire knowledge about PFDs are the internet [24,27,31] and the media [30].

#### 4.1.1. Knowledge and Ideas About Pelvic Floor Anatomy and Pelvic Floor Functions

The lack of knowledge about PF anatomy and PFF among pregnant women [27,31,33] is obviously due to a lack of knowledge among women of reproductive age as a whole. In the Irish study by Falvey et al. [37], 895 female students were surveyed on their knowledge of the PF. This demonstrated a low level of knowledge about PF anatomy and PFF. For example, only 48% of participants knew that the PF consists of muscles. Only 50% of the participants were able to correctly categorize the body orifices urethra, vagina and anus as part of the PF [37]. In a Belgian study of 212 women who had not yet given birth and were not pregnant, it was shown that there was a lack of knowledge about the PF. Only 13% of respondents were able to correctly identify the number of female orifices. Only 73% were able to name at least one function of the PF, and only 20% were aware of the role of the PF in sexual function [38]. In another Brazilian study, only 20.3% of 133 women of childbearing age were able to name various functions of the PF [39].

Kammers et al. discussed, that even in the modern age, a shameful, conservative, lack of sex education or the historically evolved view of a female ‘sinful’ body underpins a low or rudimentary knowledge of female PF and PFF. They emphasize the importance of an enlightened sexu-al and educational policy, starting in schools and continuing during pregnancy, in order to preventively promote sexual and pelvic floor health [33]. Furthermore, suitable prenatal education programmes for pregnant women are considered necessary. These should in-clude explicit anatomy and function of the PF, but also possible pregnancy-associated PF changes, in order to promote the PF health of pregnant women and preventively counter-act PFD [27,31].

#### 4.1.2. Basic Knowledge of Pelvic Floor Dysfunctions (UI, POP, FI) in the Context of Pregnancy and Birth

The lack of basic knowledge about PFD among pregnant women [23,24,25,26,27,28,29,30,31,32] is also reflected in a general lack of knowledge among women of reproductive age. The results of a Spanish study showed in their survey of 768 female students that overall knowledge of PFDs was moderate. The overall PIKQ score was 14 out of 24 points to be achieved (14 ± 6.919). The participants showed significantly better UI skills compared to POP (8.89 ± 2.861 vs. 5.19 ± 4.543) (*p* < 0.001) [40]. Freitas et al. were also able to show in their study that UI and POP were only known to 25.6% of the participants. With regard to their possible treatment options, only 23.3% were able to name them correctly [39]. The influence of pregnancy and childbirth on developing PFDs appears to be underestimated overall. Neels et al. showed that the risk factor for a possible developing UI, POP, or FI was correctly assigned by only 9% of the participants in their study population of 212 women.

Most participants were aware of the risk of pregnancy and childbirth, and only associated these with the risk of UI [38].

#### 4.1.3. State of Knowledge on the Prevention of Pelvic Floor Dysfunction or on Measures to Restore Pelvic Floor Health in the Presence of Pregnancy-Associated Pelvic Floor Dysfunction

The lack of basic knowledge on the prevention of PFD or restoration of pelvic floor health in pregnant women [26,27,29,30] is also reflected by a general lack of knowledge among women of reproductive age. Falvey et al. showed that 83.9% of their study population rated preventive PFMT as important, but 57.6% of women had never performed PFMT before, and only 6% of participants practiced PFMT regularly [37]. Neels et al. showed that 92.7% of their study population did not feel sufficiently informed about the overall context of the PF and PFD. Furthermore, 92.8% of the participants did not know what PFMT was, and 97.1% had never performed PFMT [38].

#### 4.1.4. Factors That Support an Improvement in Pregnant Women’s Knowledge of the Pelvic Floor and Possible Pregnancy-Associated Pelvic Floor Dysfunction

Pregnant women cite the internet as the main source of knowledge about PFD [24,27,31]. All authors also reported that the internet as a source of knowledge does not significantly improve knowledge about the PF and PFD [24,27,31]. Hill et al. pointed out that non-English-speaking study participants used the internet significantly less than those who spoke English at home [27]. Tennfjord et al. showed overarching and unspecified media as the main source used for contextualized knowledge acquisition by pregnant women, although there was also a low overall knowledge of UI and POP in this study [30]. Overall, the authors listed criticized the fact that on the one hand, the internet is a preferred medium for acquiring knowledge, which appears to have a higher status than healthcare professionals, but at the same time the content presented does not appear to be sufficient to generate adequate knowledge of PFM or PFD for pregnant women [24,27,30,31].

Books are used as an important source of information about pregnancy and birth [24] and also for information about PFM [27] and PFD [24,27,31]. O’Neill et al. reported that pregnant women with a high level of education, in particular, generate context-related information from books. At the same time, they had significantly better knowledge of UI and POP [24]. Hill et al. showed something similar and also found that books are mainly used by English-speaking pregnant women to acquire knowledge about PFM. In comparison, pregnant women who were not usually English-speaking used them significantly less [27].

In their study, Hill et al. explicitly pointed out that speaking and understanding the respective national language significantly increases knowledge about PFM compared to women who do not speak it [27]. A study from Denmark shows a general trend that pregnant migrant women in various validated surveys have both lower e-health literacy and health literacy compared to women of Danish origin. The researchers do not see the reason for this in the lack of motivation of pregnant migrant women to engage with the internet or e-books. Rather, ethnic minorities as a whole appear to face greater challenges in terms of language barriers combined with a lack of ability to use digital technology, which affects their e-health literacy [41].

Family members and friends are further sources of information for pregnant women [24,30,31], but do not necessarily lead to an increase in knowledge about UI and POP in the context of pregnancy and birth [24].

The finding that pregnant women who work as healthcare professionals have a higher level of knowledge about UI and POP [23,32] is consistent with that of Pizzoferrato et al. [42]. Here, medical professionals (MF) were asked about their knowledge of PFM and PFD compared to women in the general population (n = 856). The authors found that around half of the women surveyed without a medical specialism had a significant lack of knowledge about PFM and PFD. Thus, the medical professional (MF) background appears to be an effective protective factor against a lack of knowledge in this context [42].

Specialist care during pregnancy is associated with better knowledge of pelvic floor dysfunction in three studies. Farihan et al. reported that pregnant women who received antenatal specialist care had better knowledge of UI and POP (*p* = 0.000) than pregnant women who received care from medical officers or health care trainees [25]. Mckay et al. showed that pregnant women who have already been treated by a urologist or urogynaecologist tend to have a higher level of knowledge about POP [23]. Yohay et al. reported significantly higher knowledge of UI and POP when pregnant women had already seen a urologist/urogynaecologist (*p* = 0.004). Furthermore, pregnant women who had previously shown UI symptoms and had already received treatment were significantly more knowledgeable about UI [32]. DeLancey et al. and Hübner et al. [43,44] also emphasized the importance of sufficient specialist knowledge with regard to possible pregnancy-associated changes in PF. They recommend that pregnant and postnatal women receive adequate knowledge transfer from professional groups. Only in this way can they be empowered to make informed decisions regarding their pelvic floor health in the overall context of pregnancy and birth, and at the same time be provided with the necessary therapeutic measures. The specific transfer of knowledge on the PF and PFD by gynaecological and urogynaecological specialists is an important basis for strengthening women’s health literacy [43,44].

Kammers et al. suggest that knowledge regarding the anatomical structure of the PF could only be improved through open sexual education and sex education in schools and in families. Even in the modern age, a shameful, conservative outlook and lack of sex education, or even the historically grown view of a female ‘sinful’ body, underpins a low or rudimentary knowledge of the female PF and PFF [33]. Further studies show that a lack of knowledge about the female PF anatomy may be due to a sense of shame on the part of both women and healthcare professionals. As a result, pregnant women or women after the birth are often not sufficiently informed or not informed at all due to shame [8,9].

Attending antenatal education (ANE) courses can significantly improve [27] or improve [31] knowledge of PFM and PFD. Hill et al. showed that ANE participants had significantly more knowledge about PFM and UI compared to pregnant women who had not participated in ANE [27]. Toprak Celenay et al. showed similar results. Pregnant women who attended ANE had a higher, but non-significant, mean knowledge of UI and POP than pregnant women who had not attended ANE [31]. In contrast, O’Neill et al. reported that in their study population, only 35% of n = 249 study participants reported attending ANE as a resource for improving their knowledge of PFD [24]. It can be assumed that ANE will be provided by midwives within the studies conducted and discussed [45,46,47]. 

However, the midwifery profession as a whole seems to be insufficiently effective as a knowledge broker in the context of PFM and PFME. According to Hill et al., midwives do not significantly improve PF health education for pregnant women within the ANE. For example, the author states that only 50% of the study participants received information on PFD, although up to 50% of the study population already reported UI. In addition, it is evident that the information provided was not appropriate for the target group, especially when pregnant women do not speak the local language (English) [27]. O’Neill et al. show a similar conclusion. Although midwives are the most frequently mentioned source of information among health professionals for pregnancy and childbirth, they do not seem to be able to improve knowledge about the PF or PFD [24]. In Turkey, the concept of ANE is not yet well established, and this also applies to the professional group of midwives who manage an ANE [45,48]. Nevertheless, the midwifery profession does not appear to contribute to increased knowledge of the PF and PFD here either [31]. The authors assume that birth and infant care were more focused upon during the ANE that took place [31]. In principle, however, ANE seems to offer a good starting point for improving pregnant women’s knowledge of PFM or PFD. ANE can be seen as a targeted educational service provided by midwives that strengthens and promotes physiological processes in the transition to parenthood and thus has a strengthening effect on health literacy [49,50]. In this context, Butterfield et al. and Terry et al. stated that the midwifery profession lacks consistent guidelines, clear communication, and evidence-based knowledge in the specific area of PF, PFME, and PFD in antenatal education (ANE) [51,52]. An important content must therefore be to convey evidence-based knowledge with regard to the promotion of PF health to pregnant women in a structured and target group-orientated way [51,52].

#### 4.1.5. Seeking Medical Help for Pregnant Women in the Event of Pelvic Floor Dysfunction

The data presented in the studies on the prevalence of PFD [25,28,29,31] corresponds to other studies that show the development of UI [3,4], FI or POP [5,53] in the context of pregnancy. The fact that pregnant women rarely seek medical help despite having PFDs [28,29,31] is explained by Liu et al. with a possible social taboo and a lack of knowledge and awareness of PFD among study participants [28]. Parlas and Bilgic reported that only 33.9% of pregnant women affected by UI symptoms (51% of study participants) sought medical help. Here too, the authors attribute this to the study participants’ lack of knowledge about PFD [29]. Toprak Celenay et al. also showed the same for UI and POP [31]. One possible reason, which has already been discussed, is that the topic of PFD is a taboo subject in the context of pregnancy and childbirth, and also fundamentally for women at all stages of life [8,9]. Further studies confirm this. UI is considered embarrassing by those affected, and therefore no medical help is sought. Overall, affected women lack contextualized knowledge [54,55].

### 4.2. Limitations

The methodology of the review was conducted without a critical appraisal of the included studies [16,18]. As shown in Table 4, various studies and study types with different data reports were included, with the presented results based almost exclusively on cross-sectional designs. The heterogeneity of the measurement instruments used in the included studies also warrants critical evaluation. For instance, tools such as the PIKQ, the PFDI-20, or self-developed, validated, and piloted questionnaires were employed to assess women’s individual knowledge of pelvic floor health. Moreover, the studies varied in sample size and setting. It is important to note that the results of this review are based on surveys conducted within globally heterogeneous healthcare systems and incorporate diverse cultural backgrounds. Although a systematic and sensitive search was carried out, it was performed on only three medically relevant databases. An increase in additional medical databases could have increased the validity of the review. Further restrictions include the exclusive search for publications in German and English in the period January 2004-July 2024. This may result in a potential language bias. Therefore, these results should be treated with appropriate caution.

### 4.3. Research Gaps and Future Directions

This scoping review, conducted in line with established methodological standards [16,17,18,21], highlights several important gaps in the current evidence on pregnant women’s knowledge of PF health. Despite a systematic and sensitive search, only a limited number of relevant studies were identified. None of the included studies originates from German-speaking countries, which limits the direct transferability of findings. However, Ayerle, and Mattern [56] emphasize that PF health is also a relevant and under-researched topic in German midwifery science, as pregnant women explicitly express a need for evidence-based support. Most studies lack socio-demographic differentiation. Factors such as education level, migration background, language skills, and socioeconomic status, which strongly influence health literacy, are rarely examined in depth [27,41]. Women with low digital or general health literacy remain underrepresented. Methodologically, the dominance of cross-sectional designs limits the depth of understanding regarding how knowledge evolves over time. Longitudinal and interventional studies are needed to evaluate the effectiveness of antenatal education (ANE) or specialist counselling [27,31]. While ANE shows some positive effects, the role of midwives as structured providers of PF education remains unclear and inconsistently implemented [24,27,31,51,52]. Qualitative research is also scarce, although several studies point to emotional and cultural barriers such as shame and taboo [8,9,33]. The increasing use of digital media by pregnant women as a primary source of information [24,27,30,31] raises concerns about the quality and accessibility of online content, especially for vulnerable groups. Finally, interdisciplinary perspectives are missing. Although specialist involvement is considered important [23,25,32,43,44], responsibilities among healthcare professionals are often undefined. Educational science, sociology, and psychology should be more strongly integrated into future research to enable comprehensive and culturally sensitive educational approaches.

### 4.4. Implications for Midwifery Science and Broader Practice

The findings of this scoping review clearly highlight the pivotal role of midwives in promoting pelvic floor health throughout pregnancy and the postpartum period. Midwives are uniquely positioned to support physiological processes and prevent pregnancy-related disfunctions through early risk detection, targeted education, and individualized interventions. However, the evidence shows that pregnant women generally have limited knowledge about PF anatomy, PF muscles (PFM), and pelvic floor dysfunctions (PFD), including urinary incontinence (UI), pelvic organ prolapse (POP), and fecal incontinence (FI) [23,24,25,26,27,28,29,30,31,32,33].

To address these knowledge deficits, midwives must make full use of their unique position as easily accessible experts for pregnant women to ensure a structured, evidence-based antenatal education. Relevant formats should be employed to provide fundamental information on PF anatomy and function, physiological changes during pregnancy, and the risk of pregnancy- and birth-related PFD. Preventive pelvic floor muscle training (PFMT) should be a standard component of this education. Antenatal education settings should systematically incorporate content on pelvic floor health, including basic anatomy, preventive behaviours, exercises to reduce the risk of incontinence, and information on treatment options. Participatory and culturally sensitive teaching methods, such as anatomical models, visual aids, and the use of plain language, have proven particularly effective, especially for women with low health literacy and women with a migrant background [57].

Combining verbal education with body-centered training, such as guided pelvic floor exercises, can enhance the effectiveness of information delivery. Web-based platforms should be evidence-based, interactive, and easily accessible. Recommended features include multilingual modules, practical videos, self-assessment checklists, and intuitive navigation to support users with low digital health literacy. Moreover, integrating expert-verified educational tools into prenatal care, such as apps, videos, or web modules developed by interdisciplinary teams, can help ensure that pelvic floor training websites are scientifically sound. Mobile health applications offering reminders and training instructions can further improve adherence and learning outcomes [58]. Overall, a combination of personalized midwife-led counselling and digitally supported education appears to be the most effective approach for sustainably improving PF knowledge during pregnancy and the postpartum period.

Where possible, midwives should also assess PFM function (e.g., via vaginal palpation and the Oxford score) to identify weakness or dysfunctions early and to initiate individualized support [59,60].

Furthermore, early life-stage interventions are warranted. Educational efforts on PF health should begin before pregnancy, ideally during adolescence. Current NICE guidelines recommend introducing girls and young women, starting around age 12, to the concept of PF health and preventive PFMT [61,62]. Midwifes could play a critical role in implementing such early education, which is urgently needed in heterogeneous global school and healthcare systems [63]. For example, by expanding existing initiatives like “midwives in schools” in Germany [64]. This may also help destigmatize PF-related topics and facilitate open discussions about sexual and reproductive health.

Given the increasing number of pregnant women with migration backgrounds, educational strategies must also be culturally sensitive and linguistically accessible. For migrant populations, it is crucial to consider cultural background, language barriers, and varying perceptions of the body and health. This should be supported through community-based access points, native-language facilitators, or cooperation with migrant organizations. Further initiatives could also be taken in the clinical setting, e.g., target-group-orientated protocols for shared care between primary care providers and public maternity hospitals, or even a commitment by hospitals to quality initiatives in the context of support for migrant women. The provision and use of interpreting services in clinical and non-clinical prenatal care should be a matter of course in order to address specific issues such as PF health appropriately and individually. Through supported interpersonal communication, women with a migration background can be enabled to understand important health information or develop adherence to treatment [57,65]. Although antenatal education is a reimbursable service in Germany [66] there is currently no national framework for standardized PF health content, and relevant professional guidelines lack clear assignments of responsibility [64,67]. The development of such standards, anchored in midwifery expertise, is essential.

These findings also point to broader interdisciplinary implications. The correlation between higher educational levels and greater PF health suggests a need to integrate PF topics into general health and sexuality education. From a sociological perspective, the persistence of shame and silence surrounding PFD highlights the importance of public discourse and cultural change. Psychiatrically, the psychological burden of undressed PFD, such as depression, anxiety, and social withdrawal, calls for collaboration with mental health professionals in prevention, education, and care pathways.

## 5. Conclusions

This scoping review synthesized existing evidence on the health literacy of pregnant women regarding pelvic floor anatomy, function, and dysfunctions. Across all included studies, a substantial knowledge gap was identified. No relevant data were found for Germany or German-speaking countries. But the findings can reasonably be applied to these contexts. These results emphasize the need for structured, targeted, and standardized education on PF health, delivered by midwives and integrated into both antenatal care and broader public health strategies. Midwives are ideally placed to lead this work, provided they are supported by national guidelines, institutional frameworks, and interprofessional cooperation. Educational content should be tailored to the literacy and cultural background of women, ensuring equal access to essential health knowledge. This review also reinforces the value of early education on PF health, particularly for girls and young women. Global inequalities in educational access, as documented by UNICEF [68], continue to limit opportunities for health promotion among adolescent females, especially in low-income countries. Addressing these disparities is critical for long-term improvements in women’s reproductive health. Pregnancy should be understood as a “teachable moment”, a time when women are particularly receptive to health information and motivated to engage in preventive behaviours [69,70]. Leveraging this period through coordinated educational strategies can have a lasting impact on women’s PF health, improve quality of life, and reduce the long-term burden of pelvic floor dysfunctions.

## Figures and Tables

**Table 1:** PCC framework.

P	Population	pregnant women
C	Concept	knowledge
C	Context	pelvic floor and pelvic floor disorders

**Table 2:** Search strategy.

PubMed Search Strings: Listed According to PCC		Results	Date
#1 P	“pregnanc*” [Title/Abstract] OR “Pregnant Women” [Title/Abstract] OR “Pregnant Woman” [Title/Abstract] OR “Pregnancy” [MeSH Terms] OR “Pregnant Women” [MeSH Terms]	1,158,974	29 July 2024
#2 C	“level of awareness” [Title/Abstract] OR “knowledge pregnant women” [Title/Abstract:~2] OR “Health Literacy” [MeSH Terms] OR “health knowledge, attitudes, practice” [MeSH Terms]	140,771	29 July 2024
#3 C	“Pelvic Floor” [Title/Abstract] OR “Pelvic Floor” [MeSH Terms] OR “Pelvic Floor Disorders” [MeSH Terms]	14,824	29 July 2024
#4 total-term	(“Pelvic Floor” [Title/Abstract] OR (“Pelvic Floor” [MeSH Terms] OR “Pelvic Floor Disorders” [MeSH Terms])) AND (“pregnanc*” [Title/Abstract] OR (“Pregnant Women” [Title/Abstract] OR “Pregnant Woman” [Title/Abstract]) OR (“Pregnancy” [MeSH Terms] OR “Pregnant Women” [MeSH Terms])) AND (“level of awareness” [Title/Abstract] OR “knowledge pregnant women” [Title/Abstract:~2] OR (“Health Literacy” [MeSH Terms] OR “health knowledge, attitudes, practice” [MeSH Terms]))	46	29 July 2024
CINAHL Search Strings: listed according to PCC		Results	Date
#1 P	(MH “Pregnancy+”) OR (MH “Expectant Mothers”) OR TX (pregnancy* OR “pregnant women”)	300,841	31 July 2024
#2 C	(MH “Health Literacy”) OR (MH “Health Knowledge”) OR TX (“health literacy” OR (health N2 knowledge) OR (level N2 awareness))	62,505	31 July 2024
#3 C	(MH “Pelvic Floor Muscles”) OR (MH “Pelvic Floor Disorders”) OR TX “pelvic floor”	6208	31 July 2024
#4 total-term	((MH “Pregnancy+”) OR (MH “Expectant Mothers”) OR TX (pregnanc* OR “pregnant women”)) AND ((MH “Health Literacy”) OR (MH “Health Knowledge”) OR TX (“health literacy” OR (health N2 knowledge) OR (level N2 awareness))) AND ((MH “Pelvic Floor Muscles”) OR (MH “Pelvic Floor Disorders”) OR TX “pelvic floor”)	17	31 Juli 2024
Web of Science Core Collection Search String: listed according to PCC		Results	Date
#1 P	TS = (pregnanc* OR “pregnant women” OR “pregnant woman”)	610,786	05 August 2024
#2 C	TS = ((Health NEAR/2 (Knowledge OR literacy)) OR Awareness)	2,409,636	05 August 2024
#3 C	TS = (“pelvic floor*”)	16,216	05 August 2024
#4 total-term	((TS = (pregnancy* OR “pregnant women” OR “pregnant woman”)) AND TS = (Knowledge OR “health literacy” OR Awareness)) AND TS = (“pelvic floor*”)	138	05 August 2024

**Table 3:** Inclusion criteria.

Articles addressing the aspects of PCC (pregnant women, knowledge, pelvic floor, and pelvic floor disorders)
Articles in English and German languages
Articles published January 2004 onwards to 31. July 2024

**Table 4:** Analyzed studies.

Author(s)	Year	Title	Country	Objektive	Population/Sample Size	Setting	Methodology	Screening Tool(s) and Outcome Measures
Mckay et al. [23]	2019	Knowledge of Pelvic Floor Disorders in Obstetrics	USA	This study aimed to investigate the knowledge and demographic factors associated with a lack of knowledge about UI (POP) in pregnant and postpartum women.	Pregnant women in all trimesters of pregnancy or women within 8 weeks postpartum *n* = 399 nulliparity: 33.2% primiparity: 33.2% multiparity (>2): 32.4% postpartum women: 6.8% mean age 28.5 ± 6.0 years	Women were recruited at 9 locations, including outpatient clinics, private practices, and hospital-based antenatal, labour and delivery units. Each of them was tied together with three major hospitals: Yale New Haven Hospital, Yale New Haven Bridgeport Hospital, and Danbury Hospital/ Western Connecticut Health Network.	A multicentre, cross-sectional survey statistical tests: descriptive presentation in mean and standard deviation and/or percentages using logistic regression; multivariate logistic regression	PIKQ; additional PFDI-20
O’Neill et al. [24]	2016	Knowledge of pelvic floor problems: a study of third trimester, primiparous women	UK	This study aims to explore the knowledge of PFD in pregnant primiparous women in the 3rd trimester.	Primiparous pregnant women in the third trimester of pregnancy; *n* = 249 Mean gestational age: 33 ± 5 weeks mean age 30 ± 6 years;	The study took place in 3 maternity hospitals in London, UK.	A multicentre, cross-sectional study with survey statistical tests: mean and standard deviation; multivariable linear regression	Using a self-designed, validated questionnaire (based on PIKQ)
Farihan et al. [25]	2022	Prevalence, Knowledge, and Awareness of Pelvic Floor Disorder among Pregnant Women in a Tertiary Centre, Malaysia.	Malaysia	This study aimed to assess the knowledge and awareness about the PF and PFD among pregnant women in a tertiary Centre in Malaysia. In addition, the relationship between women’s risk factors regarding their knowledge and awareness of the PF should be evaluated.	Nulliparous and multiparous pregnant women; gestational age > 18 weeks *n* = 424 primiparity: 33.3% multiparity: 66.7%; median of gestational age: 36.1 (32.0, 38.2) weeks; median age: 31.5 (29.0, 35.0) years	The study took place in a maternity centre in Malaysia. All included pregnant women were interviewed on site using questionnaires.	Cross-sectional study with survey statistical tests: Chi2 test; mean and standard deviation/median and interquartile range	PIKQ; additional PFDI-20
Geynisman-Tan et al. [26]	2017	Is Something Missing From Antenatal Education: A Survey of Pregnant Women’s Knowledge of Pelvic Floor Disorders.	USA	This study aimed to describe the knowledge of PFD among a cross-section of pregnant women.	Nulliparous and multiparous pregnant women; gestational age > 18 weeks; *n* = 402 primiparity: not listed multiparity: not listed mean age ± SD: 34 ± 6 years	The study was carried out in New York Presbyterian Prenatal Department Hospital. During their waiting period, the women included were interviewed by research assistants using a questionnaire.	Cross-sectional survey study statistical tests: Kruskal–Wallis test; Wilcoxon signed-rank test; paired knowledge questions were analysed by McNemar test.	PIKQ
Hill et al. [27]	2017	Pregnant women’s awareness, knowledge, and beliefs about pelvic floor muscles: a cross-sectional survey,	Australia	The aims of the study were to evaluate pregnant women’s levels of awareness, knowledge, and beliefs about the PFMs and PFMEs.	Nulliparous and multiparous pregnant women; *n* = 633 primiparity: 50.1% multiparity: 48.2% mean gestational age: 28.7 ± 7.8 weeks mean age: 29.2 ± 5.3 years	The study was conducted in various health facilities of the Department of Health, Western Australia (DoHWA). Included women were able to complete the survey on site and online.	Cross-sectional survey study statistical tests: Cross-tabulations of Chi2 tests; Fisher’s exact tests	Using a self-designed, validated, and piloted questionnaire with outcome measures awareness of PFMs and knowledge of PFMs and PFMEs, and the role of PFMs as a key function in preventing UI
Liu et al. [28]	2019	Knowledge of pelvic floor disorder in pregnancy.	Singapore	The aims of this study were to assess the level of knowledge about pelvic floor disorders among pregnant women in a local population.	Nulliparous and multiparous pregnant women in the third trimester of pregnancy; *n* = 104 nulliparity: 50.0% multiparity: 50.0% mean gestational age: 34.4 ± 3.8 (28–41) mean age 30.6 ± 5.0 years;	The study was conducted at the KKWomen’s and Children’s Hospital maternity clinics using self-administered questionnaires.	Cross-sectional study with survey statistical tests: one-way ANOVA analysis; nonparametric tests	Using a self-designed, validated 47-item questionnaire with outcome measures, knowledge of PFD (UI/POP/FI), and how they relate to pregnancy and childbirth
Parlas and Bilgic [29]	2024	Awareness of urinary incontinence in pregnant women as a neglected issue: a cross-sectional study	Turkey	The aim of this study is to determine the UI awareness of pregnant women and their knowledge and attitudes in this context.	Pregnant women in all trimesters of pregnancy *n* = 255 nulliparity: 42% primiparity: 38% multiparity: 20% gestational age of included women: first trimester: 7.5% second trimester: 32.9% third trimester: 59.6% mean age 29.2 ± 5.1 years	Face-to-face interview in a university hospital in Turkey.	A cross-sectional study with survey statistical tests: descriptive statistical methods; Independent samples t-test, one-way ANOVA, Pearson’s correlation test	PIKQ: whereby only the survey on UI was carried out; Additionally, UIAS
Tennfjord et al. [30]	2023	Pelvic Floor Disorders and Pelvic Floor Muscle Exercise: A Survey on Knowledge, Attitude, and Practice among Pregnant Women in Northwest Ethiopia	Ethiopia	The aim of the study was to examine POP and UI as well as the knowledge, attitudes, and practices of PFME. Furthermore, the connection of these factors with parity in pregnant women in Gondar, Ethiopia.	Pregnant women in all trimesters of pregnancy; *n* = 502 nulliparity: 26.5% multiparity: 73.5% mean age 28.1 + 6.2 years	Data collection was carried out in 7 randomly selected antenatal care health centres in the central Gondar zone (Gondar City; Wogera; Dembia Gondar Zuria) using face-to-face interviews.	A facility-based cross-sectional study with survey statistical tests: regression models	PIKQ; additional questions about knowledge, attitude, and practice to PFME
Toprak Celenay et al. [31]	2021	Do community-dwelling pregnant women know about pelvic floor disorder?	Turkey	The study aimed to assess knowledge and awareness about pelvic floor disorders (PFDs) in pregnant women. Whether knowledge about PFDs depends on gestational age, parity, participation in an ANE or history of UI and/or POP was also examined	Pregnant women in all trimesters of pregnancy *n* = 241 nulliparity: 78.2% multiparity: 21.8% median gestational age: 21 (4–40) weeks mean age 29.03 + 4.66 years;	Face-to-face setting at two gynaecology and obstetrics clinics, including a private clinic and a training and research hospital,	A cross-sectional descriptive study with survey statistical tests: mean and standard deviation; median; Mann–Whitney U test or Kruskal–Wallis test; KR-20 coefficient	PIKQ; Three additional targeted questions were used to assess pregnant women’s awareness of UI and POP
Yohay et al. [32]	2022	Knowledge of women during the third trimester of pregnancy regarding pelvic floor disorders	Israel	The aim of this study was to objectively assess the knowledge regarding pelvic floor disorders (PFDs) among women during the third trimester of pregnancy.	Pregnant women in the third trimester of pregnancy. *n* = 649 nulliparity: 26.3% multiparity: 73.7% Hebrew: 405 Arabic: 244; median gestational age: not listed weeks mean age 30.48 ± 5.91 years	Large university medical centre in Israel	A cross-sectional study with survey statistical tests:using mean and standard deviation, t-test or one-way ANOVA; calculation of the Pearson’s or Spearman’s correlation coefficient.	PIKQ
Kammers et al. [33]	2021	Knowledge and perceptions of pregnant women about the reproductive system	Brazil	To understand the perception of some adult Brazilian pregnant women in relation to the importance attributed to the biological body from the perspective of the reproductive system and the PF during pregnancy.	Nulliparous, primiparous, and multiparous pregnant women in all trimesters of pregnancy; *n* = 13 mean age: not listed	The study took place at the time of the antenatal visit in the waiting room of the health centres of the city of Florianópolis/Santa Catarina. Interviews and graphic preparations were carried out in a private room adjacent to the waiting room.	Qualitative study with semi-structured interviews	Using a semi-structured interview guide and a graphical representation of the reproductive system and pelvic floor, and complementary sociodemographic data

**Table 5:** Important results and derived recommendations.

Author(s)	Important Results	Derived Recommendations
Mckay et al. [23]	Results showed low knowledge regarding UI and POP in included women. A total of 74.2% of included women showed a lack of knowledge proficiency about UI, and 70.6% showed a lack of POP knowledge proficiency. Overall, 49.7% of included women knew that childbirth was a risk factor for UI, and 29.2% knew that childbirth was a risk factor for POP. Among symptomatic women who reported UI, approximately 41% did not know that childbirth was a risk factor for their symptoms. After adjustment, Hispanic women, primiparous women, and women with lower levels of education were significantly more likely to lack knowledge proficiency regarding UI. The final model for POP knowledge showed that women with a high school diploma or less were more likely to lack POP knowledge compared to women with a bachelor’s degree or higher. Women who had previous visits to a urologist or urogynecologist were less likely to lack POP knowledge. Pregnant women working in a medical field were less likely to lack UI and POP knowledge compared to those who did not (ORs: 0.26 [95% CI: 0, 13–0.52] and 0.38 [95% CI: 0.21–0.70]). Pelvic floor disorder during pregnancy was common; 39% of pregnant women acknowledged having UI; 4.8% experienced symptoms of POP.	The results of the study show that an effective educational strategy for risk reduction and the inclusion of women from socio-economic backgrounds with higher risk is necessary to expand knowledge about PFM and PFD. Standardized educational sessions by healthcare professionals to discuss issues related to PFDs and their treatment options during pregnancy are recommended. The focus should be on the range of interventions, including behavior modification, pelvic floor exercises, and use of pessaries, to improve women’s quality of life.
O’Neill et al. 2016 [24]	Results showed low knowledge regarding UI, POP, and FI in the included pregnant women. The average knowledge score across all areas was low at 45%. The average composite knowledge score was the highest in the domains of UI (63%), but low when questions covered more detailed knowledge levels (41%, further POP (36%), and the lowest knowledge score in the domain of FI (35%). There is a positive association between knowledge scores and education to a tertiary level, where knowledge scores were 18% lower in women educated to mid-secondary school level than in those educated to tertiary/degree level. The most commonly cited information sources on pregnancy and delivery used by the respondents were the internet (84%), books (82%), further sources were also the midwife (72%), doctor (65%), and ANE, only 35%.	An important source of knowledge is healthcare professionals (doctors and midwives). They should take their task more seriously to address pelvic floor problems early in pregnancy and provide information about them to make it easier for women to access information. There is also potential for improvement in the content conveyed in the ANE. The lack of a positive relationship between the level of knowledge and the use of the most popular source of information—the internet—points out that there is room for improvement in online content.
Farihan et al. 2022 [25]	Results showed low knowledge regarding UI and POP in the included pregnant women. A total of 80.4% of included pregnant women had a low level of knowledge regarding UI, and 45.0% had a low level of knowledge about POP. The median total score for knowledge about pelvic floor disorders was 12 points, which was considered low (cut-off point of 16). The median knowledge score PIKQ UI was 7 points, and the median knowledge score PIKQ POP was 6 points. Having a tertiary level of education and receiving antenatal specialist care were associated with better knowledge regarding PF, UI, and POP (*p* = 0.000). Pelvic floor disorder during pregnancy was common; 46.1% of included pregnant women had at least one symptom of PFD during pregnancy, 62.7% experienced symptoms of UI, 41.8% experienced symptoms of POP, and 37.8% experienced symptoms of FI.	In principle, increasing levels of education and prenatal specialist medical care are associated with better knowledge of the pelvic floor and possible dysfunctions. A fundamental new educational program to bridge knowledge gaps about PFD in pregnant women and women is necessary.
Geynisman-Tan et al. 2017 [26]	Results showed poor knowledge regarding UI and POP in the included pregnant women. The mean ± SD knowledge score for PIKQ UI was 7.9 points (66 ± 12% correct answers). The mean ± SD knowledge score for PIKQ POP was 4.9 points (41 ± 17% correct answers). Pregnant women were more likely to know that delivery could result in incontinence (62%) than pelvic organ prolapse (42%; *p* = 0.02). At least 83% of pregnant women knew that pelvic floor exercises can prevent urinary incontinence. There is a lack of knowledge about exercises to prevent POP. Only 55% of study participants correctly answered: “Certain exercises can help prevent POP from getting worse”; *p* < 0.001.	Although pregnant women often have pelvic floor problems with a high risk of pelvic floor dysfunction and therefore increasingly consult doctors, they still do not have adequate knowledge about pelvic floor dysfunction. Prenatal care, education, and patient empowerment must be improved here. Future studies focused on providing information about improving pelvic floor health are needed for women and pregnant women about PFDs and their possible treatment focus during pregnancy and after birth.
Hill et al. 2017 [27]	The findings show a very limited knowledge in the included pregnant women about the anatomy and function of the PFMs, further UI, and PFMEs. Although 76% of included women knew that PFMs can prevent UI, 27% of them knew that they prevented FI. A total of 41% of included pregnant women thought it was normal to leak urine when pregnant. Only 5.4% of included pregnant women were able to correctly assess the anatomy of the PF, and 20.7% could not identify any PFM function. Only 11% of the included pregnant women practiced PFMEs. Study participants who had ANE (28%) were significantly more knowledgeable about pelvic floor function (*p* < 0.001). Included pregnant women who did not speak English at home (18%) were significantly less knowledgeable about PFMs and PFMEs (*p* < 0.001), and significantly less likely to have attended, or planned to attend, ANE classes (*p* < 0.001). The main source of knowledge for the included pregnant women to acquire knowledge about PFM and PFMEs is the internet.	A deeper educational approach and further research appear necessary to further expand the awareness and knowledge of PFMs among pregnant women at all levels of education. ANE is an important basis for promoting pelvic floor health. Pregnant women need more health education regarding PFMs. Education should be diverse, especially for women with a migrant background. Further research is needed to effectively expand comprehensive methods of providing ANE to pregnant women to increase their knowledge and awareness of PFMs. They should be supported in developing their skills and motivation to engage in PFMEs.
Liu et al. 2019 [28]	Results showed low knowledge regarding UI and POP in the included pregnant women. The knowledge score for UI was the highest (mean score of 46.2% ± 0.3). The knowledge score for FI was 39.8% ± 0.3 and for POP was 35.3% ± 0.3. Mean knowledge scores increased significantly with educational level (*p* = 0.046) and age (*p* = 0.021). There is a lack of knowledge about and prevention of UI; 40% of included women do not know that PFMEs in pregnancy can help to prevent UI after childbirth. Pelvic floor disorder during pregnancy was common; 31.7% of included women reported experiencing urinary incontinence, 2.9% of respondents reported POP, and 0.96% had FI. None of the women sought medical attention or treatment for these conditions.	The level of knowledge about the pelvic floor and possible dysfunctions highlights the importance of educating young women of reproductive age about protecting their pelvic floor health. It is important to reevaluate public education campaigns on prenatal care to empower women of childbearing age to make more informed decisions about performing pelvic floor exercises to reduce the risk of future pelvic floor dysfunction.
Parlas and Bilgic 2024 [29]	Results showed low knowledge about UI among pregnant women. Only 6.3% of the participants correctly answered all of the items in the UI knowledge scale; the mean score of PIKQ UI was 8.07 ± 2.64. A total of 44% did not know what to do to prevent and manage UI. More than half of the pregnant women (62.8%) had not heard about PFME, and most of them (83.5%) did not perform PFME. The mean UIAS score was 42.33 ± 3.48. The results showed a positive correlation between UI knowledge and attitude value (r = 0.35, *p* = 0.00). Pelvic floor disorder during pregnancy was common. The prevalence of UI was 51% during pregnancy.	There is an urgent need for education about UI and women’s attitudes towards UI. Pregnant women should be educated about UI and its symptoms and how to manage it. They should be encouraged to visit health services, nurses, and midwives for early counselling on UI. Specific health education and counselling services for women should be developed and standardised within multidisciplinary teams. Pregnant women should be encouraged to attend antenatal classes where PFME is taught practically and knowledge is improved using educational materials and pictures.
Tennfjord et al. 2023 [30]	Overall, the entire study population showed insufficient knowledge of POP and UI. A total of 76.7% of nulliparous women had good knowledge of POP, and 74.4% had good knowledge of UI. Only 44.4% of nulliparous women had good knowledge of PFME, and 51.1% had good knowledge of attitudes toward PFME. A total of 93.2% of nulliparous women did not perform PFME. Of the multiparous women, only 41.2% had good knowledge of PFME, and 45.8% had good knowledge of attitudes toward PFME. A total of 92.7% of multiparous women did not perform PFME. After adjusting for all covariates, educational level was significantly associated with better knowledge of PFME (*p* < 0.001).	Most pregnant women used ANC services throughout their pregnancy. Despite this, study participants’ knowledge of UI and POP was poor, as was their attitude and practice towards PFD, indicating a need to improve the quality of healthcare services.
Toprak Celenay et al. 2021 [31]	Overall, the entire study population showed a very low knowledge of POP and UI. The median PIKQ-UI was 6 points, and PIKQ-POP scores were 5 (Scales 0–12). Overall, 92.3% of the included women lacked knowledge regarding UI and 57.5% regarding POP. A total of 18.9% of pregnant women participated in ANE. The median PIKQ-UI and PIKQ-POP scores were higher in women who had attended ANE. A total of 45% of the included pregnant women cited the internet as the main source of information for disseminating knowledge about UI and POP. The healthcare professional doctor (20%) or midwife (8.6%) was less considered a source of information. Pelvic floor disorder during pregnancy was common. Of the included pregnant women, 18.6% had UI and 3.6% had sudden onset of POP.	To promote pelvic floor health, comprehensive prenatal education programs on UI and POP, as well as PMFT by health care professionals, are an essential necessity for all pregnant women. These should not only teach birth and infant care, but also explicitly address the topics of PFM, UI, and POP. Even women who are not pregnant should be informed about PFM, POP, and UI.
Yohay et al. 2022 [32]	Overall, the entire study population showed a low knowledge of POP and UI. The average PIKQ scores were 7.65 ± 2.8 and 5.32 ± 2 for UI and POP, respectively. There were significantly higher average scores in UI and POP noted among health care workers (UI: 10.19 ± 2.3 vs. 7.34 ± 2.6, *p* < 0.001; POP: 8.27 ± 2.7 vs. 4.97 ± 2.6, *p* < 0.001), age over 35 was associated with significantly higher scores (8.13 ± 2.8 vs. 7.48 ± 2.8; *p* = 0.008) in PIKQ-UI and (5.75 ± 2.5 vs. 5.19 ± 2.9; *p* = 0.025) in POP. Women with an academic degree show a significantly higher level in PIKQ-UI and POP (*p* < 0.001) than those without an academic degree. This also applied to pregnant women with higher incomes (*p* < 0.001).	Research shows low knowledge of PFD among women in the third trimester of pregnancy in Israel. Pregnancy offers good timing for education and interventions. From this, it can be deduced that targeted educational programs should be created for defined target groups in order to improve knowledge about PFD in the relevant population and thus improve the quality of life of women.
Kammers et al. [33]	There is a lack of knowledge about the anatomy and function of the female PFM. Various specific subject areas are reflected in the results. The female body is usually discovered during puberty. A conservative upbringing leads to a lack of knowledge about one’s own femininity/female body/reproductive organs. The pelvic floor is often associated with structures such as the vaginal canal and labia and is less easily imaged. The uterus, ovaries, and fallopian tubes are known to participants as a complex reproductive system in the lower abdomen and can also be represented graphically. The pelvic floor is less well known as part of the female body; its structure seems hidden from the imagination of the participants and is therefore difficult to represent.	The results of this study show the importance of school and its sex education and health education programs. To maintain sexual health and avoid illnesses or risks related to pregnancy, women need sufficient knowledge about their bodies. It is clear that in many cultures women’s bodies or their sexuality are still not openly discussed, leading to a lack of knowledge. While organs such as the ovaries, fallopian tubes and uterus are largely known, other elements such as the pelvic floor and vagina are unknown and hidden and are more likely to be considered shameful.

Abbreviation: attended antenatal education (ANE); antenatal care (ANC); fecal incontinence (FI); health care professionals (HCP); pelvic floor (PF); Urinary Incontinence Attitude Scale (UIAS); pelvic floor disorder (PFD); Pelvic Floor Distress Inventory-20-Questionnaire (PFDI-20); pelvic floor muscles (PFMs); pelvic floor muscle exercises (PMFEs); Prolapse and Incontinence Knowledge Questionnaire (PIKQ); pelvic organ prolapse (POP); urinary incontinence (UI).

## Data Availability

Not applicable.
